# Supplementary material for: Fatal cytokine release syndrome by an aberrant FLIP/STAT3 axis
Source: Cell Death Differ. 2021 Sep 13;29(2):420–38. doi: 10.1038/s41418-021-00866-0 (PMC8435761; doi:10.1038/s41418-021-00866-0)
Supplement: Supplementary file 1 — Supplementary material [file 41418_2021_866_MOESM1_ESM.doc]

SUPPLEMENTAL INFORMATION

Supplemental Figures

- Fig. S1. Clinical characteristic of enrolled patients and immunosuppressive function of monocytes.
- Fig. S2. vFLIP chimera mice develop cytokine release syndrome.
- Fig. S3. Systemic and local features of cytokine release syndrome in vFLIP mice.
- Fig. S4. Single-cell transcriptional profiling of lung-infiltrating cells in vFLIP mice and BAL-derived immune cells obtained from COVID-19 patients.
- Fig. S5. Characterization of the pharmacological STAT3-targeting effectiveness in vFLIP mice.
- Fig. S6. Characterization of the in vivo STAT3-silencing effectiveness in vFLIP mice.
- Dataset 1. scRNA-seq data and statistics.


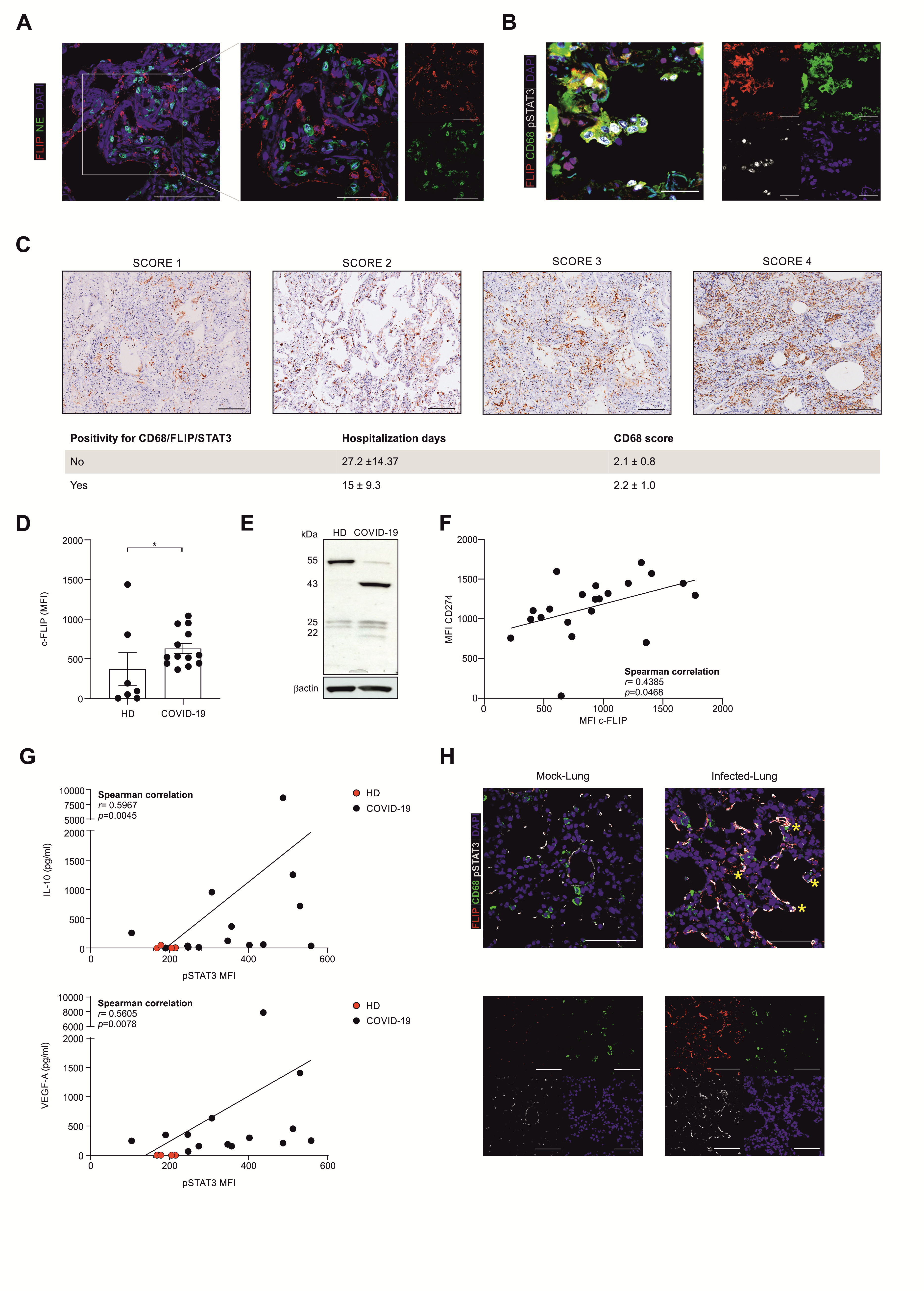


Fig. S1. Clinical features of enrolled patients and immunosuppressive function of monocytes and CD68^+^ cells in lung of hACE2 infected mice.

A Representative immunofluorescence (IF) staining of lung-infiltrating neutrophils (NE^+^ cells) in COVID-19 patients. Cells were stained for NE (green), FLIP (red) and DAPI (blue). Scale bar, 50 μm. B Representative immunofluorescence (IF) staining of monocytes (CD68^+^ cells) of COVID-19 patient. Cells were stained for CD68 (green), c-FLIP (red), pSTAT3 (white) and DAPI (blue). Scale bar, 20 μm. C CD68^+^ cells infiltration in lung samples (n=23) of the enrolled COVID-19 patient’s cohort was defined using an arbitrary scoring from 1 to 4. Representative IHC images used to define CD68 score. The score of CD68-expressing cells does not discriminate between patients with a shorter (15±9.3 days) or a longer (27.2±14.37 days) hospitalization time. D FLIP expression in circulating monocytes (CD14^+^ cells) purified from health donor (HD, n=7) or COVID-19 patients (n=13). E Western blot analysis for FLIP expression (FLIP_L_, p43, FLIP_S_ and p21) on circulating monocytes (CD14^+^ cells) isolated from HD or COVID-19 patients. F Correlation between CD274 (PD-L1) and c-FLIP expression in COVID-19 circulating monocytes (CD14^+^ cells) (n=19). G Correlation between the release of IL-10 or VEGF-A cytokines and pSTAT3 expression in circulating monocytes (CD14^+^ cells) from HD (red, n=4) and COVID-19 patients (black, n=13). H Representative indirect immunofluorescence (IFA) staining of FLIP (red), CD68 (green), pSTAT3 (white) and DAPI (blue) in lungs of HFH4-hACE2 transgenic mice SARS-CoV-2-infected or mock-infected. Scale bar, 60μm. Correlation analysis was performed by Spearman’s rank correlation (F, G). Data are reported as mean ± S.E.M. *p ≤ 0.05, **p ≤ 0.01 and ***p ≤ 0.001 by Mann–Whitney test (D).


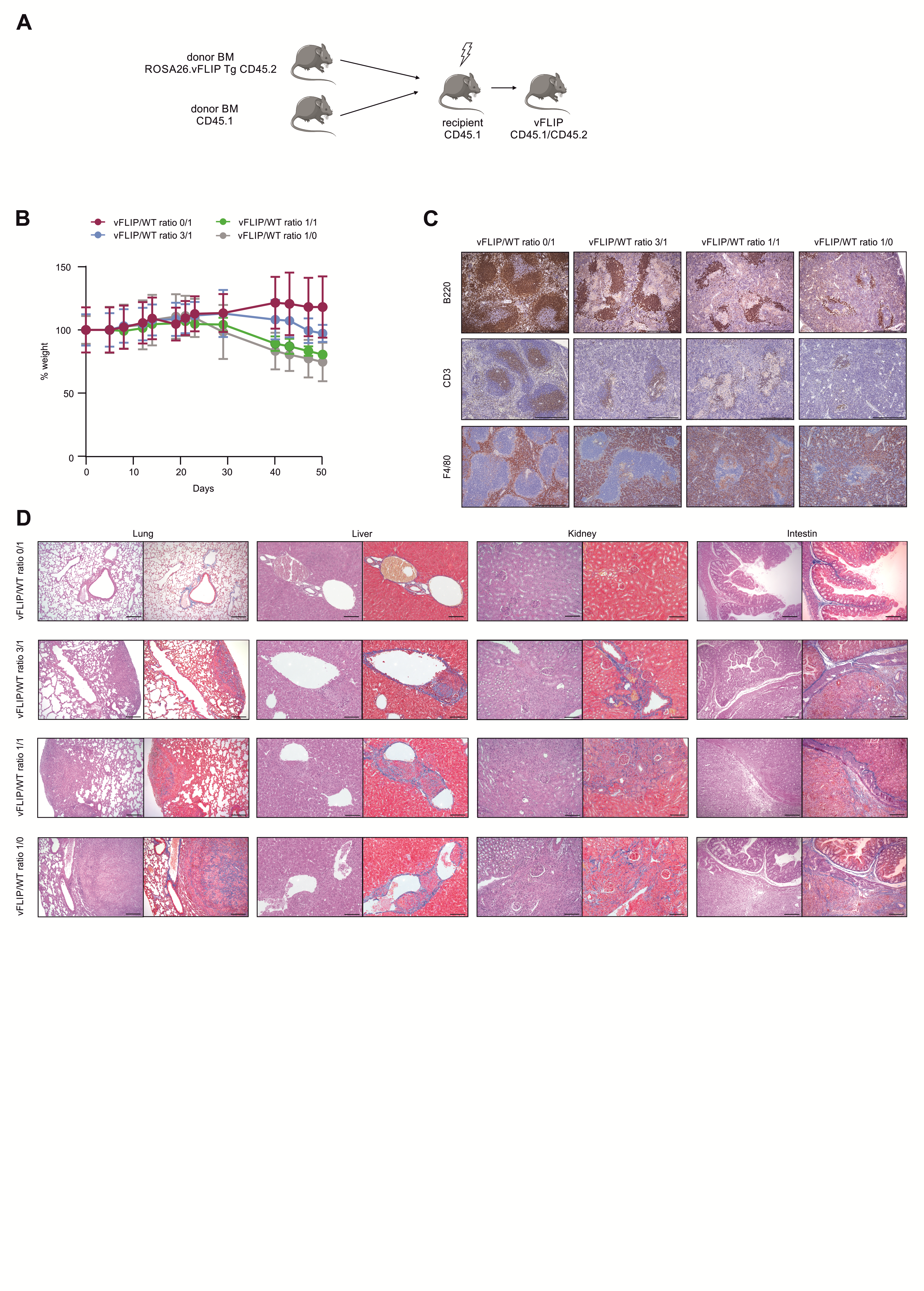


Fig. S2. vFLIP chimera mice develop cytokine release syndrome.

A Experimental layout of chimera (vFLIP) mice generation. B Variation of body weight during CRS progression in vFLIP chimera mice (n=3, for each condition). C IHC analysis of spleen in vFLIP mice: lymphocytes (B220^+^ cells: B lymphocytes; CD3^+^ cells: T lymphocytes) and mononuclear phagocytes (F4/80^+^ cells). Scale bar, 400 μm. D Representative H&E-stained microscopy images and Masson’s Trichrome of lung, liver, kidney and intestine of vFLIP mice. Scale bar, 100 μm.


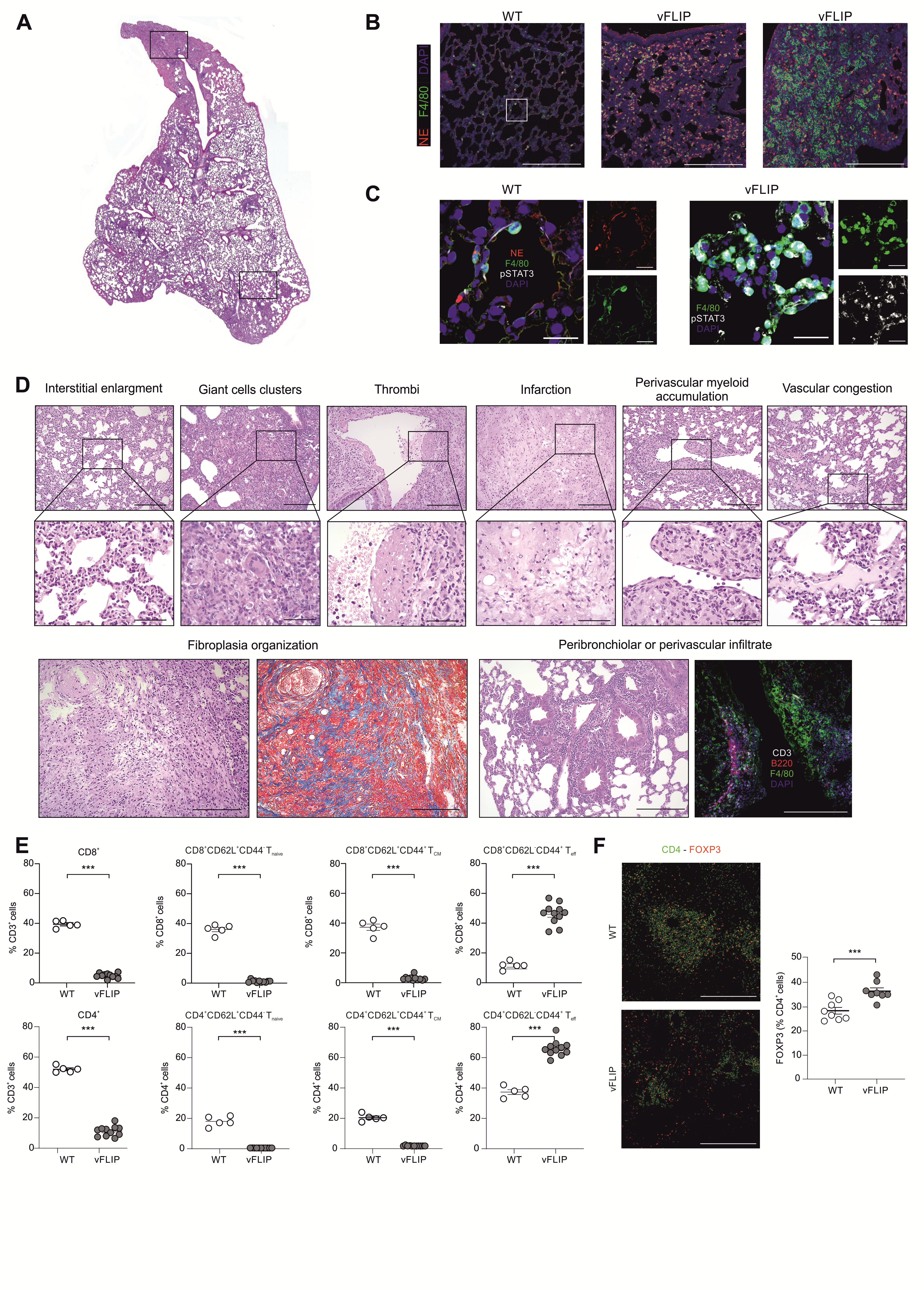
Fig. S3. Systemic and local features of cytokine release syndrome in vFLIP mice.

A Representative H&E-stained microscopy of lung tissue of vFLIP mice. B Representative IF staining of lung-infiltrating neutrophils (NE^+^ cells) and mononuclear phagocytes (F4/80^+^ cells) in WT or vFLIP mice. Cells were stained for DAPI (blue), neutrophil elastase (NE) (red, middle panel) and F4/80 (green, right panel). Scale bar, 200 μm. C Representative IF staining of lung-infiltrating neutrophils (NE^+^ cells) and mononuclear phagocytes (F4/80^+^ cells) in WT or vFLIP mice. Scale bar, 20 μm. Cells were stained for DAPI (blue), NE (red) or F4/80 (green) and pSTAT3 Tyr705 (grey). D Representative H&E-stained microscopy images of pathological score: interstitial enlargement, giant cells clusters, thrombi, infarction, perivascular myeloid accumulation, vascular congestion, fibroplasia organization and peribronchiolar or perivascular infiltrate vFLIP mice. Scale bar, 200 μm (upper panel) and 50 μm (bottom panel). E Flow cytometry analysis of CD3^+^ T cell subsets in the spleen of vFLIP mice (n=11) and WT mice (n=5). Lymphocytes were segregated into T_effector_ (CD62L^-^CD44^+^), T_naive_ (CD62L^+^CD44^-^) and T_central memory_ (CD62L^+^CD44^+^). F Representative IF staining of CD4^+^ FOXP3^+^ cells in spleen of vFLIP mice (n=8) and WT (n=8). Data are reported as mean ± S.E.M. *p ≤ 0.05, **p ≤ 0.01 and ***p ≤ 0.001 by Mann–Whitney test (E, F).


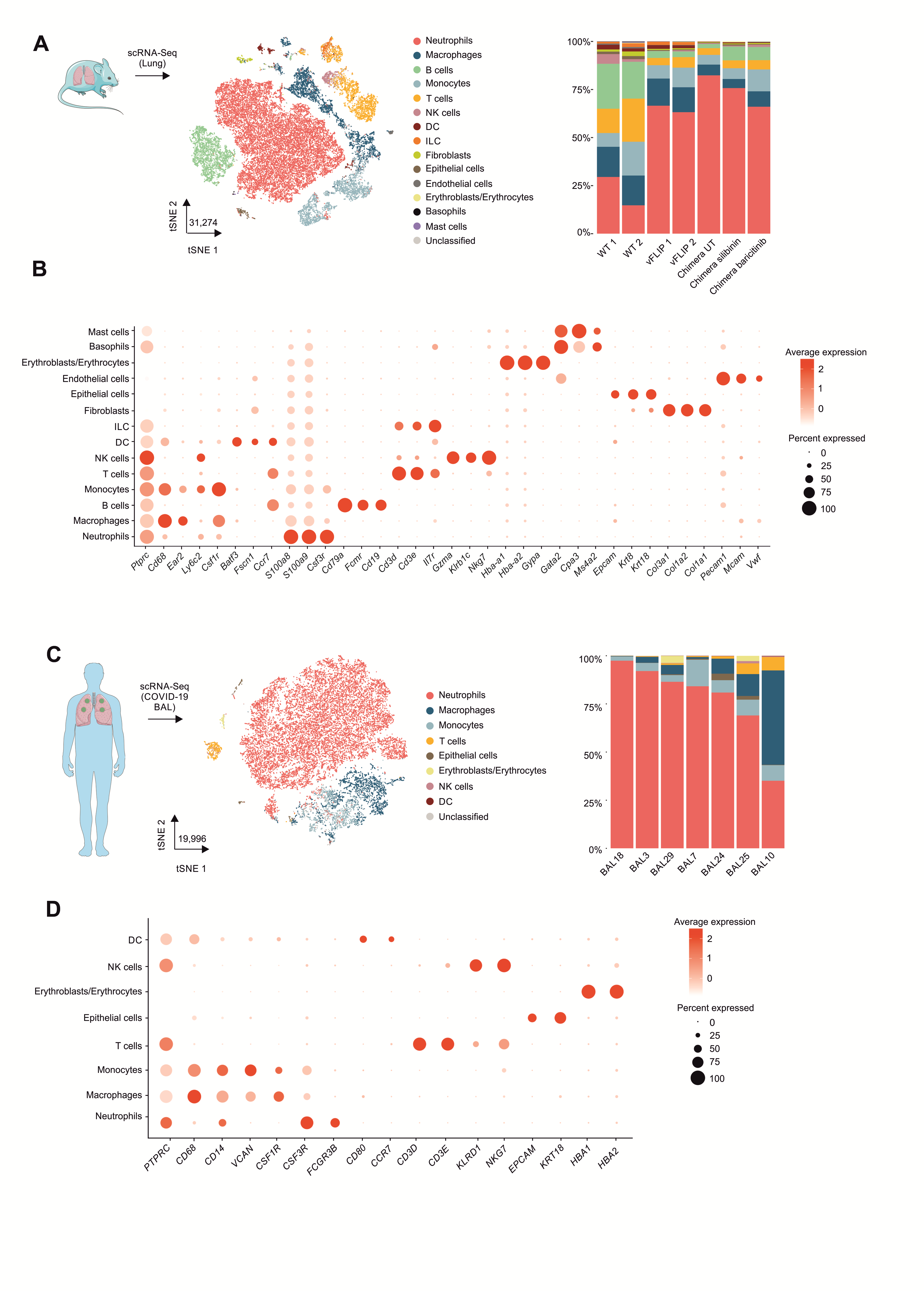


Fig. S4. Single-cell transcriptional profiling of lung-infiltrating cells in vFLIP

mice and BAL-derived immune cells obtained from COVID-19 patients.

A tSNE representation of scRNA-seq from all mouse samples (31,274 cells) colored according to cell type. Stacked bar plots representing cell type proportions across all the mouse samples. B Dot plot showing the scaled average expression of known marker genes for the mouse cell populations identified. C tSNE representation of scRNA-seq from fatal COVID-19 BALs patients (19,996) colored according to cell type. Stacked bar plots representing cell type proportions across all the human BAL samples. D Dot plot showing the scaled average expression of known marker genes for the cell types identified in the BALs.


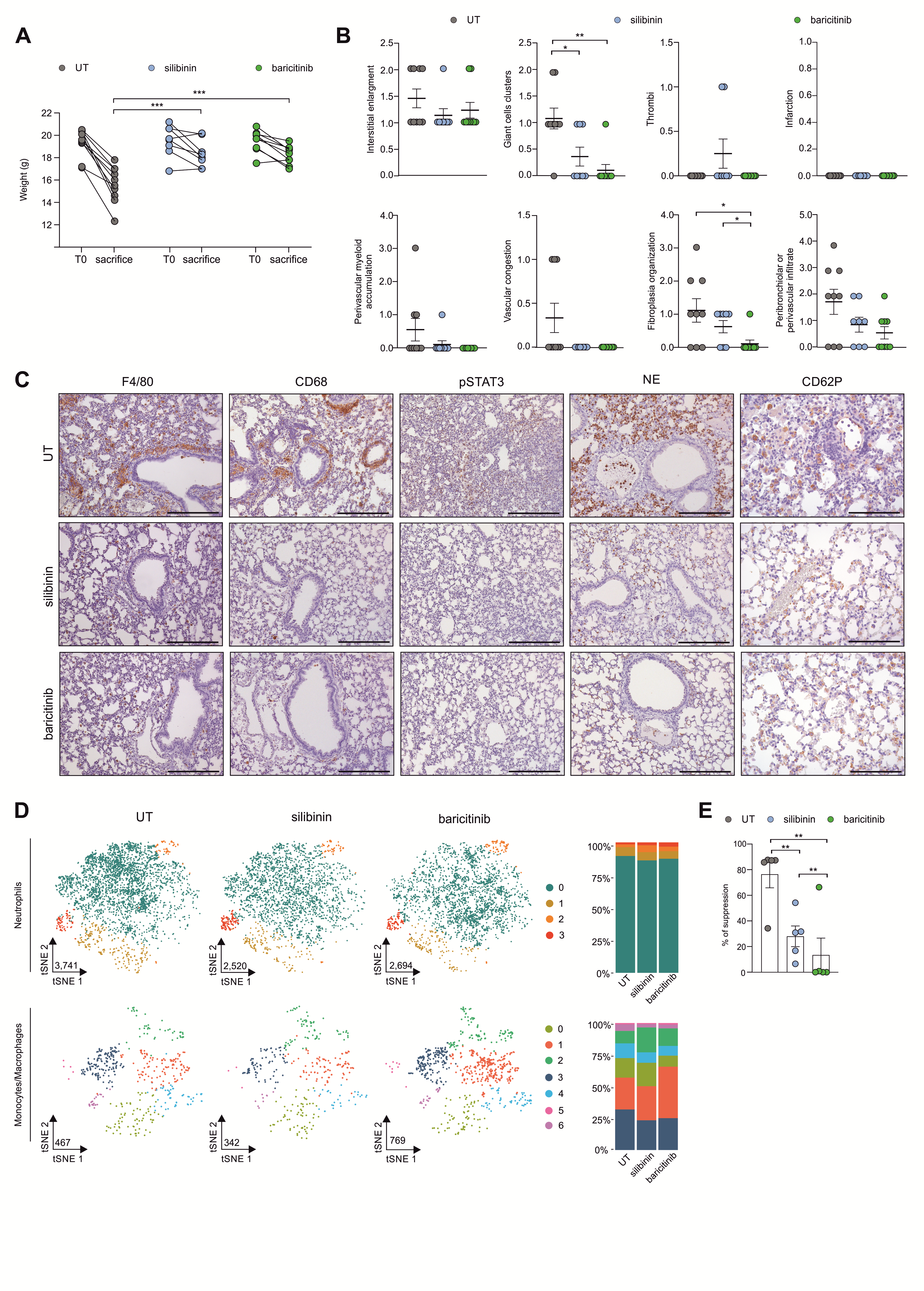


Fig. S5. Characterization of the pharmacological STAT3-targeting effectiveness

in vFLIP mice.

A Body weights of vFLIP chimera mice before treatment (T0) or at the end of treatment (untreated, n=9; silibinin, n=8; baricitinib, n=9). B Dot plots of pathological score parameters: interstitial enlargement, giant cells clusters, thrombi, infarction, perivascular myeloid accumulation, vascular congestion, fibroplasia organization and peribronchiolar or perivascular infiltrate (untreated, n=9; silibinin, n=8; baricitinib, n=9). C Representative IHC images of F4/80, CD68, p-STAT3, NE and CD62P in lungs isolated from vFLIP mice untreated or treated with either silibinin or baricitinib. Scale bar, 200 μm. D tSNE representation of neutrophil subsets across untreated (3,741) mice and treated with silibinin (2,520) and baricitinib (2,694) colored according to cluster analysis (top). Stacked bar plots representing neutrophil clusters proportions across conditions. tSNE representation of monocytes/macrophages subsets across untreated (467) mice and treated with silibinin (342) and baricitinib (769) colored according to cluster analysis (bottom). Stacked bar plots representing monocytes/macrophages cluster proportions across conditions. E Functional assay performed at 1:3 ratio of PBMCs:CD14+ cells using purified monocytes from COVID-19 patients (n = 5) treated with silibinin (200μm), baricitinib (200μm) or left untreated. Data are reported as mean ± S.E.M. *p ≤ 0.05, **p ≤ 0.01 and ***p ≤ 0.001 by Mann–Whitney test (A,B,E).


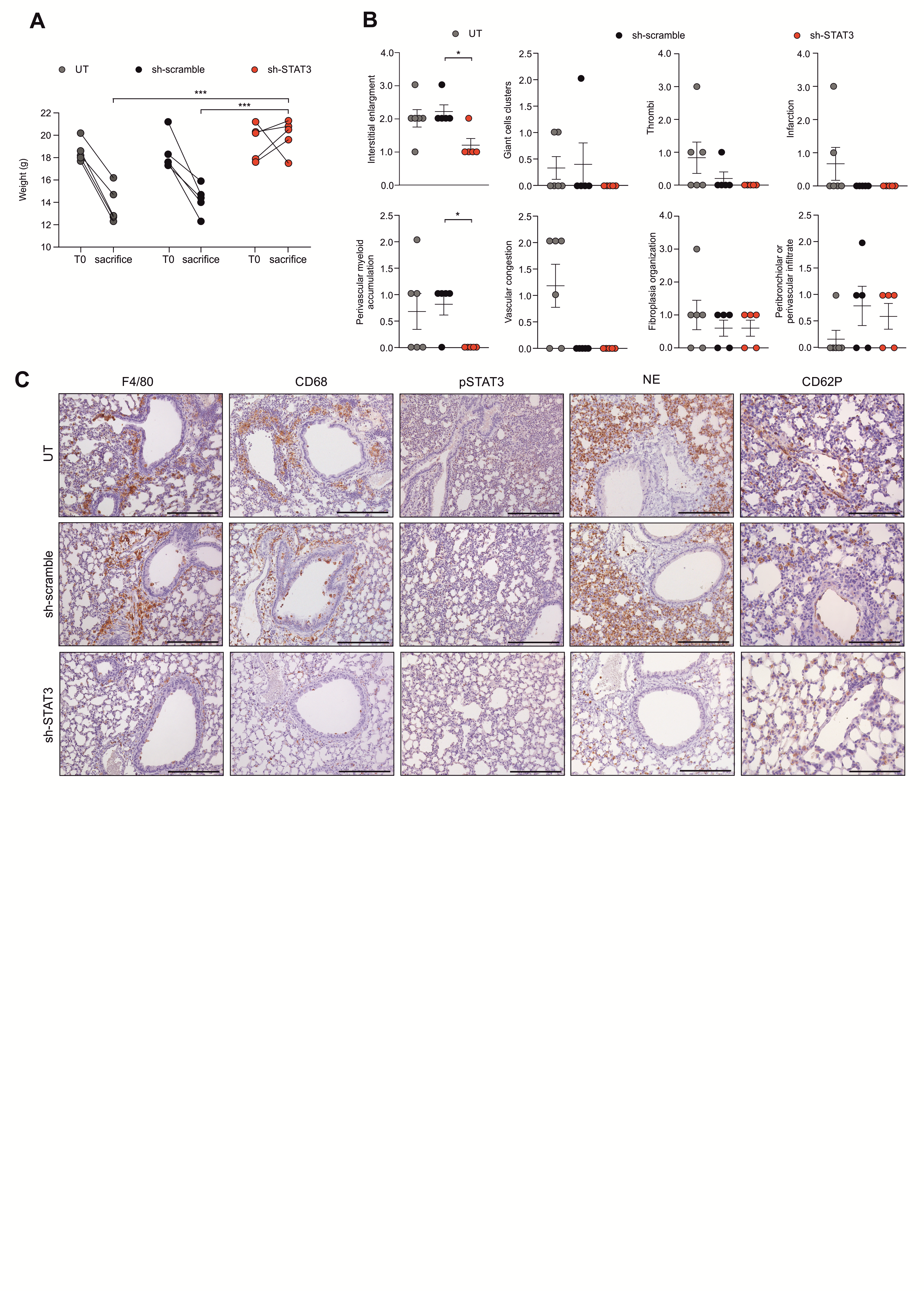
Fig. S6. Characterization of the in vivo STAT3-silencing effectiveness in vFLIP

mice.

A Body weights of vFLIP chimera mice before treatment (T0) or at the end of treatment (untreated, n=6; sh-scramble, n=5; sh-STAT3, n=5). B Dot plots of pathological score parameters: interstitial enlargement, giant cells clusters, thrombi, infarction, perivascular myeloid accumulation, vascular congestion, fibroplasia organization and peribronchiolar or perivascular infiltrate (untreated, n=6; sh-scramble, n=5; sh-STAT3, n=5). C Representative IHC images of F4/80, CD68, p-STAT3, NE and CD62P in lungs isolated from vFLIP mice untreated or treated with either sh-STAT3 or sh-scramble. Scale bar, 200 μm. Data are reported as mean ± S.E.M. *p ≤ 0.05, **p ≤ 0.01 and ***p ≤ 0.001 by Mann–Whitney test.
